# Innervation of the Human Cavum Conchae and Auditory Canal: Anatomical Basis for Transcutaneous Auricular Nerve Stimulation

**DOI:** 10.1155/2017/7830919

**Published:** 2017-03-15

**Authors:** P. Bermejo, M. López, I. Larraya, J. Chamorro, J. L. Cobo, S. Ordóñez, J. A. Vega

**Affiliations:** ^1^Walden Medical Neuro Digital Therapies S.L., Gijón, Spain; ^2^Servicio de Cirugía Máxilofacial, Hospital Universitario Central de Asturias, Oviedo, Spain; ^3^Departamento de Morfología y Biología Celular, Grupo SINPOs, Universidad de Oviedo, Oviedo, Spain; ^4^Clinica Ordoñez, Oviedo, Spain; ^5^Facultad de Medicina y Ciencias de la Salud, Universidad Autónoma de Chile, Temuco, Chile

## Abstract

The innocuous transcutaneous stimulation of nerves supplying the outer ear has been demonstrated to be as effective as the invasive direct stimulation of the vagus nerve for the treatment of some neurological and nonneurological disturbances. Thus, the precise knowledge of external ear innervation is of maximal interest for the design of transcutaneous auricular nerve stimulation devices. We analyzed eleven outer ears, and the innervation was assessed by Masson's trichrome staining, immunohistochemistry, or immunofluorescence (neurofilaments, S100 protein, and myelin-basic protein). In both the cavum conchae and the auditory canal, nerve profiles were identified between the cartilage and the skin and out of the cartilage. The density of nerves and of myelinated nerve fibers was higher out of the cartilage and in the auditory canal with respect to the cavum conchae. Moreover, the nerves were more numerous in the superior and posterior-inferior than in the anterior-inferior segments of the auditory canal. The present study established a precise nerve map of the human cavum conchae and the cartilaginous segment of the auditory canal demonstrating regional differences in the pattern of innervation of the human outer ear. These results may provide additional neuroanatomical basis for the accurate design of auricular transcutaneous nerve stimulation devices.

## 1. Introduction

The direct cervical stimulation of the vagus nerve at the cervical level was approved a few years ago by the Food and Drug Administration of USA as a viable alternative for the treatment of adult and adolescent epilepsy refractory crisis [1]. Thereafter, it was used with variable success in the treatment of several neurological and nonneurological diseases [2, 3]. Currently, there is evidence obtained from human and experimental studies that intermittent and chronic stimulation of the vagus nerve can be effective for the treatment of epilepsy and depression [4, 5]. Nevertheless, because this is an invasive method that requires surgery, substitutive strategies to stimulate the vagus nerve transcutaneously have been proposed [6, 7].

One of these alternatives is the transcutaneous stimulation of the auricular branch of the vagus nerve (ABVN), also known as Alderman's nerve or Arnold's nerve. This method has proved to be effective for the treatment of depression [8–11], epilepsy [12–14], headache [15, 16], or autism disorders [17] and has potential use in the treatment of multiple sclerosis, Alzheimer's disease [18], Parkinson's disease [19], and dystonias [20]. Moreover, it was protective for cerebral ischemia in a rat model [21].

Most of the commercially available devices for transcutaneous ABVN stimulation are applied on the concha of the pinna. However, this auricular region is supplied not only by ABVN, but also by the auriculotemporal nerve and contributions of the glossopharyngeal [22], facial [23], and cervical nerves (lesser occipital nerves and* auricularis maior*) [24–26]; importantly, all these nerves contribute to the innervation of the external auditory meatus and external auditory canal, and only the cymba conchae is regarded to be exclusively innervated by ABVN [27]. Therefore, this region is innervated by a mix of nerves including fibers of the ABVN [28]. Thus, the precise knowledge of external ear innervation seems to be of maximal interest to accurately design devices for transcutaneous auricular stimulation. The present study was designed to map the precise localization of nerves in the human cavum conchae* (cavum conchae auricula)* and the cartilaginous segment of the auditory canal (*meatus acusticus externus cartilagineus*, MAEC), using histology and immunohistochemistry to identify nerves.

## 2. Materials and Methods

### 2.1. Materials

Eleven outer ears, including the pinna* (auricula)* and MAEC, were removed form 6 frozen Spanish cadavers (Area de Anatomía y Embriologia Humana, Departamento de Morfología y Biología Celular, Universidad de Oviedo, Spain) of both sexes (3 males and 3 females) with ages ranging from 66 to 84 years. The material was obtained in compliance with Spanish Laws. The pieces were washed with tap water for 12 h, fixed in 4% formaldehyde for 48 h, washed with tap water again for 12 h, and divided into samples containing the cavum conchae and the MAEC separately. Thereafter, the pieces were processed for routine paraffin embedding. The pieces were sectioned 10 *µ*m thick perpendicularly to the longitudinal axis of the pinna and MAEC, respectively, and the sections were processed for standard Masson's trichrome staining and immunohistochemistry.

### 2.2. Immunohistochemistry

Indirect peroxidase-antiperoxidase immunohistochemistry was carried out as follows: sections were rinsed in 0.05 M HCl Tris buffer saline (TBS; pH 7.5) containing 0.1% bovine serum albumin and 0.1% Triton X-100. The endogenous peroxidase activity (3% H_2_O_2_) and nonspecific binding (10% foetal calf serum) were blocked, and the sections were incubated overnight in a humid chamber (relative humidity: 85–90%) at 4°C with the primary antibodies: mouse anti-neurofilament protein (NFP; clone 2F11; Dako, Glostrup, Denmark; prediluted), mouse anti-S100 protein (S100P; clone 4C4.9; Thermo Scientific, Freemont, CA, USA; diluted 1 : 1000), rabbit anti-S100P (Dako; 1 : 1000), and mouse anti-myelin-basic protein (MBP; clone SMI 99, directed against the residues Ala-Ser-Asp-Tyr-Lys-Ser in position 131–136 of human MBP; Sternberger Monoclonals Inc., Lutherville, MD, USA; diluted 1 : 500). Then, the sections were rinsed in the same buffer as above and incubated with Dako EnVision System labelled polymer-HR anti-rabbit IgG or anti-mouse IgG (DakoCytomation, Denmark) for 30 minutes at room temperature. Finally, sections were washed and the immunoreaction was visualized using 3-3′-diaminobenzidine as a chromogen. For control purposes, representative sections were processed in the same way as described above using nonimmune rabbit or mouse sera instead of the primary antibodies, or omitting the primary antibodies in the incubation. To ascertain structural details, sections were slightly counterstained with haematoxylin and eosin.

### 2.3. Double Immunofluorescence

Sections were processed for simultaneous detection of S100 protein and MBP, in order to establish the density of myelinated nerves. Nonspecific binding was reduced by incubation for 30 minutes with a solution of 1% bovine serum albumin and the sections were then incubated overnight at 4°C in a humid chamber with a 1 : 1 mixture of rabbit anti-S100 antibody and mouse anti-MBP antibody (diluted 1 : 1000 and 1 : 500, resp.). After rinsing with TBS, the sections were incubated for 1 hour with Alexa fluor 488-conjugated goat anti-rabbit IgG (Serotec, Oxford, UK), diluted 1 : 1000, and then rinsed again and incubated for another hour with Cy™3-conjugated donkey anti-mouse antibody (Jackson ImmunoResearch, Baltimore, MD, USA) diluted 1 : 50. Both steps were performed at room temperature in a dark humid chamber. Sections were then washed, dehydrated, and mounted with Entellan®. Double staining was detected using a Leica DMR-XA automatic fluorescence microscope coupled with Leica Confocal Software, version 2.5 (Leica Microsystems GmbH, Heidelberg, Germany) and the images captured were processed using the software ImageJ version 1.43 g (McMaster Biophotonics Facility, McMaster University, Ontario; http://www.macbiophotonics.ca/). See also the legend of the Supplementary Material available online at https://doi.org/10.1155/2017/7830919.

For control purposes, representative sections were processed in the same way as described above using nonimmune rabbit or mouse sera instead of the primary antibodies or omitting the primary antibodies in the incubation. Under these conditions, no positive immunostaining was observed (data not shown).

### 2.4. Quantitative Analysis

The following parameters were determined: (a) density of NFP and S100P positive nerve profiles and (b) percentage of MBP nerve fibers (regarded as myelinated) with respect to the total number of nerve fibers (established on the basis of their expression of S100P).

*(a) Density of NFP and S100P Positive Nerve Profiles*. The density of nerve profiles in the cavum conchae and MEAC was calculated in 50 whole sections per specimen (25 sections processed for detection of NFP and 25 sections processed for detection of S100P), 50 *µ*m apart. The counts were made by two researchers independently directly under the microscope, using a 10x objective. The results (mean values ± standard deviation) were grouped as anterior and posterior for the cavum conchae and superior, anteroinferior, and posteroinferior for MAEC. Furthermore, they were divided into inside and out of the cartilage.

*(b) Percent of MBP Nerve Fibers*. To establish the percent of myelinated nerve fibers, the number of MBP-positive nerve fibers (regarded as myelinated) was determined in nerve profiles sectioned transversally (at least 10 per section) and compared with that of S100P-positive nerve fibers (regarded as the total number). The counts were made by two researchers independently directly under the microscope using a 40x objective, and the results are expressed as percentage (mean values ± standard deviation) of MBP-positive nerve fibers in each of the pinnae or MAEC mentioned above.

## 3. Results

**Figure 1:**
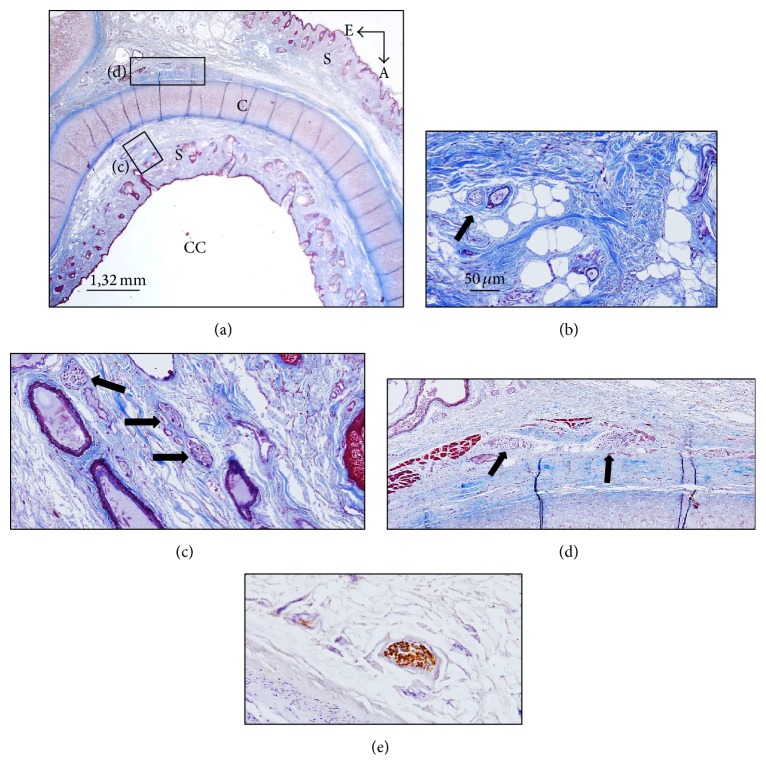
(a) Section of the cavum conchae stained with Masson's trichrome staining. (b) Small nerve trunks embedded in the subcutaneous connective tissue. (c) and (d) are enlargements of the rectangles in (a). (e) Nerve placed out of the cartilage of the cavum conchae immunostained for demonstration of S100 protein. A: anterior; E: external; CC: cavum conchae; C: auricular cartilage; S: skin. Arrows indicate nerve profiles. Scale bar is identical for (b), (c), (d), and (e).

The cavum conchae is made of fibrocartilage (auricular cartilage) covered by skin on both anterior-lateral and posterior-medial sides, and MEAC consist of cartilage continuous with the auricular one, interrupted by two or three fissures in the anterior wall, in which the cartilage is supplied by fibrous tissue. Both the cavum conchae and MEAC are lined by keratinizing stratified squamous epithelium, closely adherent to the perichondrium. The subcutaneous tissue of the cavum conchae contains fine hairs and numerous sebaceous glands, whereas at the MEAC level it has abundant hairs as well as sebaceous and ceruminous, but not eccrine sweat, glands (Figures 1(a) and 3(a)).

The nerve profiles in transverse, longitudinal, and oblique sections were clearly identified between the cartilage and the skin in the cavum conchae and between the cartilage and the skin or the pericartilaginous fibrous tissue in the MEAC. Nevertheless, intraepithelial nerve fibers or differentiated cutaneous sensory nerve formations were never observed. On the other hand, the distribution of S100P and NFP within the nerves was consistent with labeling of Schwann cells and axons, respectively, and that of MBP with myelinating Schwann cells.

### 3.1. Nerve Profiles in the Cavum Conchae

**Figure 2:**
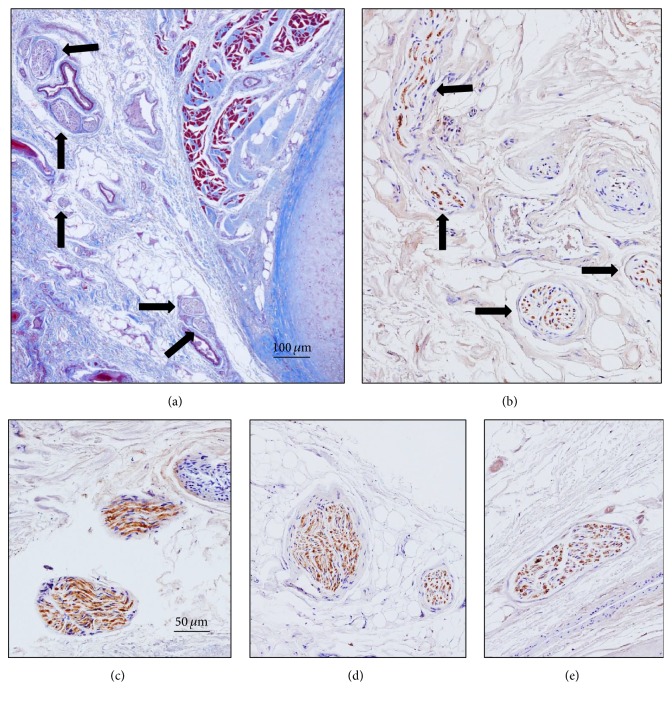
Masson's trichome staining (a) and immunostaining for neurofilaments ((b), (d), and (e)) and S100 protein (c) in sections of the cavum conchae, showing nerve profiles inside (c) and out of ((b), (d), and (e)) the auricular cartilage. Image in (a) corresponds to the auriculocephalic angle. Arrows indicate nerve profiles. Scale bar is identical for (b)–(e).

Inside the cavum conchae, small nerves were found running in the subcutaneous tissue, preferentially embedded in the fibrous tissue in the vicinity of the perichondrium (Figures 1(b) and 1(c)). In the skin covering the posteromedial side of the cartilage, nerves were localized and embedded in the fibrous tissue close to the auricular muscles (Figures 1(d) and 2(a)), primarily at the auriculocephalic angle. They displayed regular immunoreactivity for both S100P (Figures 1(e) and 2(c)) and NFP (Figures 2(b), 2(d), and 2(e); see also Figure S1 in the Supplementary Material). The density of nerves was greater in skin covering the posteromedial (28,3 ± 4,9) than the anterolateral (10,2 ± 4,7) surfaces of the auricular cartilage and the percent of MBP+ nerves fibers as well (16,1 ± 8,5% versus 8,2 ± 3,6%) (Figures 6 and 7(a)). Differences, however, were observed among the analyzed subjects that apparently were not related either with age or with gender.

### 3.2. Nerve Profiles in the MAEC

**Figure 3:**
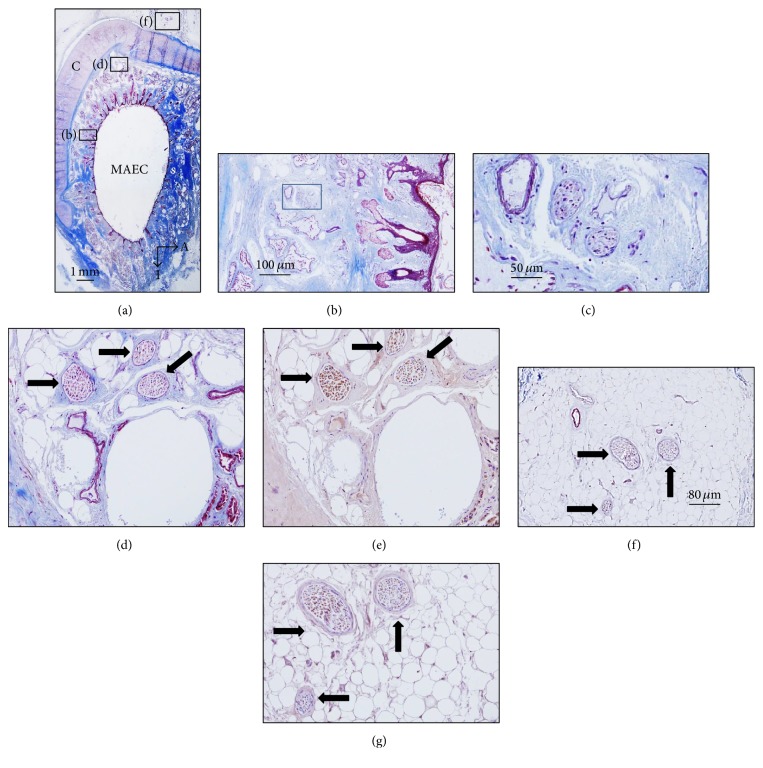
Sections of the external segment of the meatus acusticus externus cartilagineus (MAEC) stained with Masson's trichome staining ((a)–(d)) and immunostained for demonstration of neurofilaments ((e)–(g)). (b), (d), and (f) are enlargements of the square in (a). ((b), (d)) Nerves placed inside the cartilage; (f) nerves placed out of the cartilage; (c) and (g) are enlargements of (b) and (f), respectively. A: anterior; I: inferior. Arrows indicate nerve profiles. Scale bar is identical for (c), (d), (e), and (g).

**Figure 4:**
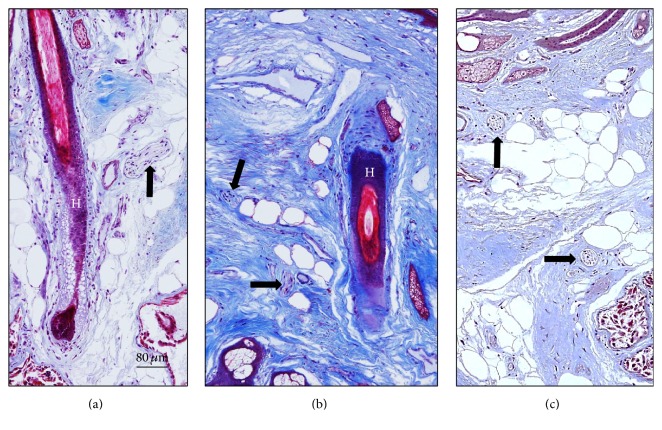
Sections of the external segment of the meatus acusticus externus cartilagineus (MAEC) stained with Masson's trichome showing details of the canal skin. H: hair. Arrows indicate nerve profiles. Scale bar is identical for (a)–(c).

**Figure 5:**
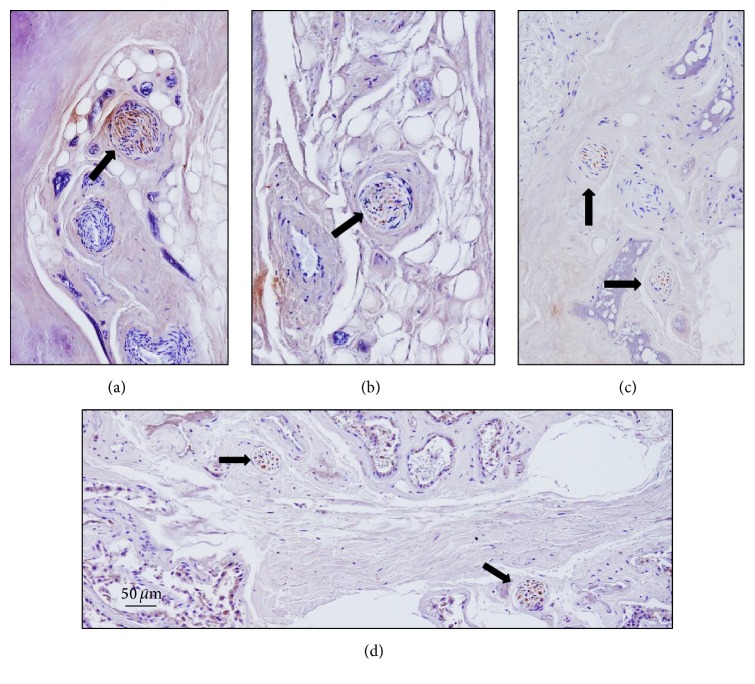
Sections of the external segment of the meatus acusticus externus cartilagineus (MAEC) showing nerve profiles out of the cartilage immunostained for S100 protein ((a), (d)) and neurofilaments ((b), (c)) stained with Masson's trichome showing details of the canal skin. Arrows indicate nerve profiles. Scale bar is identical for (a)–(d).

Likely, as in the cavum conchae, the nerves in the MAEC were localized between the skin covering the lumen of MEAC and the cartilage (Figures 3(a), 3(b), 3(c), and 3(d)) and out of the cartilage (Figures 3(f)-3(g); Figure S2 in the Supplementary Material) and as a rule nerve profiles in the MEAC were more numerous than in the cavum conchae. Between the luminal skin and the cartilage, nerves were found in the subepithelial fibrous tissue (Figures 3(a) and 3(b)), embedded in the perichondrium (Figures 3(c) and 3(d)), and occasionally in the proximity of the hair follicles (Figures 4(a) and 4(b)) and the subepidermal fibrous tissue (Figure 4(c)). Importantly, most of the nerve profiles identified were placed far off the cutaneous surface isolated from the surrounding tissues even within the cartilage (Figure 5; Figure S3 in the Supplementary Material).

**Figure 6:**
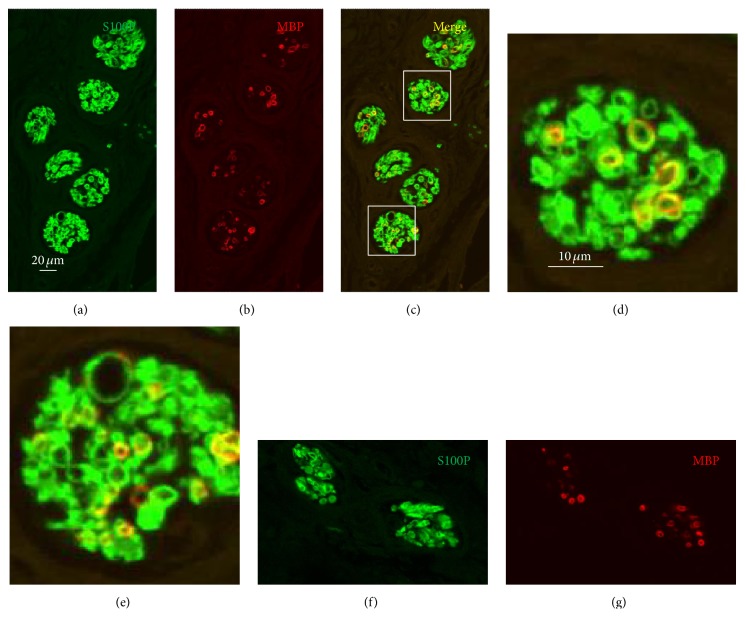
Double immunofluorescence for S100 protein (green fluorescence) and myelin-basic protein (red fluorescence) in nerves of MAEC placed out of the cartilage ((a)–(e)) or inside the auricular cartilage of the cavum conchae ((f) and (g)). Colocalization of S100 protein and myelin-basic protein results in yellow fluorescence (c); (d) and (e) are details of the square in (c). Objective 40x/1.25 oil; pinhole airy 1, XY resolution 156 nm and Z resolution 334 nm. Scale bar common for (a)–(c) and (f)-(g); scale bar common for (d) and (e).

**Figure 7:**
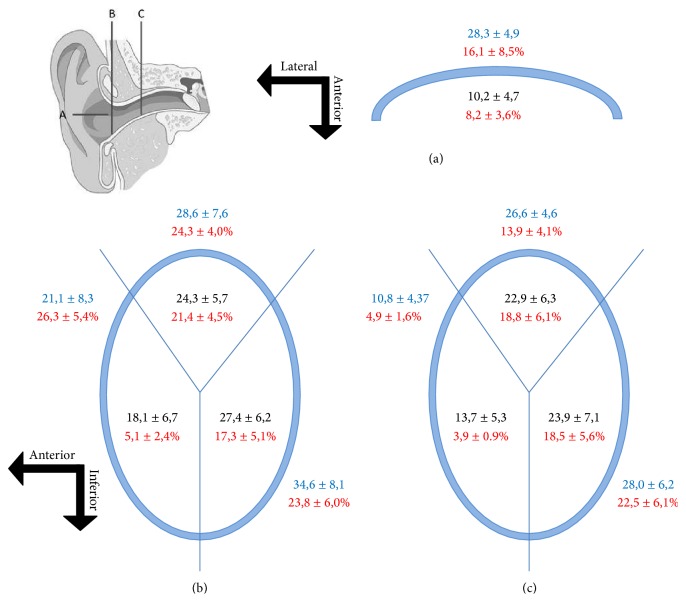
Schematic representation of the quantitative results in the cavum conchae (A) and external (B) and internal (C) segments of MAEC. (a) Data below the blue line corresponds to the anterior-lateral side of the cavum conchae, and those above the blue line correspond to the posterior-medial side of the cavum conchae. (b) Lateral segment of MAEC representing the data out of and inside the cartilage (blue line); (c) medial segment of MAEC representing the data out of and inside the cartilage (blue line). Numbers in red correspond to the percentage of myelinated nerve fibers.

The results of the quantitative study clearly demonstrated that the density of nerves was higher in the posteromedial than in the anterolateral sides of the cavum conchae and in the external surface of the MEAC cartilage with respect to the skin covering the luminal surface of the MEAC inside the cartilage (with the exception of the anteroinferior segment of the medial portion of MEAC). Also, nerve profiles were more numerously found in the superior and posterior-inferior segments of MAEC than in the anteroinferior segment. In detail, in the lateral segment of MAEC inside the cartilage, the number of nerve profiles counted was 18,1 ± 6,7 (5,1 ± 2,4% MBP+) in the anterior-inferior segment, 24,3 ± 5,7 (21,4 ± 4,5% MBP+) in the superior segment, and 27,4 ± 6,2 (17,3 ± 5,1% BMP+) in the posterior-inferior segment; out of the cartilage, the density of nerves was 21,1 ± 8,3 (26,3 ± 5,4% MBP+) in the anterior-inferior segment, 28,6 ± 7,6 (24,3 ± 4,0% MBP+) in the superior segment, and 34,6 ± 8,1 (23,8 ± 6,0% MBP+) in the posterior-inferior segment (Figures 6 and 7(b)). In the medial segment of MAEC inside the cartilage, the number of nerve profiles was 13,7 ± 5,3 (3,9 ± 0,9% MBP+) in the anterior-inferior segment, 22,9 ± 6,3 (18,8 ± 6,1% MBP+) in the superior segment, and 23,9 ± 7,1 (18,5 ± 5,6) in the posterior-inferior segment; out of the cartilage, the density of nerves was 10,8 ± 4,3 (4,9 ± 1,6% MBP+) in the anterior-inferior segment, 26,6 ± 4,6 (13,9 ± 4,1% MBP+) in the superior segment, and 28,0 ± 6,2 (22,5 ± 6,1% MBP+) in the posteroinferior segment (Figure 7(c)).

## 4. Discussion

The present study was designed to investigate the distribution of nerves in the human cavum conchae and MAEC in order to provide a detailed map that may allow the design of devices for auricular transcutaneous nerve stimulation. We have evaluated the density of nerve profiles and the percent of myelinated nerve fibers within them, without considering the cranial ganglia from which they originate since this is impossible to establish in cadaveric or surgical human material.

This study reports the relative density of nerves in human* cavum conchae* and MAEC, demonstrating that it is higher in MAEC than in the cavum conchae and in the superior and posterior MAEC walls than in the anterior one. The nerves were observed inside and out of the cartilage, their density being higher outside than inside. All the nerve profiles identified were in the dermis and the perichondrium, whereas nerve fibers directly related to or within the epidermis were never found. The thickness of the skin and its adherence to the perichondrium make it especially susceptible to the cartilage movements (including vibration) and therefore mechanical forces moving the cartilage may also stimulate the auricular cutaneous nerves. Furthermore, we established that a variable percentage of nerve fibers present in the* cavum conchae* and MAEC are myelinated. Recently, Safi et al. [29] have established the ratio of A*β* axons (measuring ≥7 *µ*m which can be regarded as myelinated nerve fibers and are those activated by direct vagus nerve stimulation [30]) with respect to the total number of fibers in the ABVN and cervical vagus nerves obtaining values of ~1 : 5 and 1 : 6 on the left and right side, respectively. Therefore, they concluded that based on these anatomical data the transcutaneous stimulation of ABVN might be an alternative to invasive stimulation of cervical vagal nerve. In our study, a variable percentage of nerve fibers in both the cavum conchae and MAEC were myelinated, therefore able to be activated by transcutaneous stimulation. Because the density of these myelinated fibers varied in the different segments of the cavum conchae and MAEC regions, stimulation techniques might consider these differences in order to increase their effectiveness.

Anatomical studies in humans have established the complexity of the innervation in outer ear zones throughout branches of ABVN, auriculotemporal nerve, and glossopharyngeal, facial, and cervical nerves [22–27]. In particular, the ABVN is especially important for the innervation of the cymba conchae [27] and the posterior wall of MAEC [24]. Experimental data in rats using retrograde tracers have confirmed the involvement of the trigeminal, facial, vagal, glossopharyngeal, and dorsal root C2–C4, and postganglionic sympathetic nerve fibers contributed to the innervation of MAE [31, 32].

It is accepted actually that electrical stimulation of sensory afferents within the auricula and MAE represents a transcutaneous manner of central nervous system activation [33], especially of the* nucleus tractus solitarii* (NTS; [34, 35]). Most of these stimuli are driven by ABVN [35–37], forming the so-called auriculovagal pathway [38], but all nerves involved in the afferent innervation of the auricula and MAE drive afferent signals, of different nature, to NTS. Therefore, although the effectiveness of the NTS stimulation might vary in relation to the nerve stimulated, it seems evident that the stimulation of any wall of the auricula and MAE can stimulate NTS or other brainstem structures. Nevertheless, differences seem to exist in the activation of NTS stimulating the anterior or posterior walls of the MAE. In fact, the stimulation of the left anterior auditory canal results in BOLD signal decreases in the area of the nuclei of the vagal nerve (which may indicate an effective stimulation of vagal afferences) while stimulation at the posterior wall leads to unspecific changes of the BOLD signal within the solitary tract [39]. But the global effects of auricular and MAE transcutaneous nerve stimulation result in activation of cerebral centers other than NTS, and presumably all together elaborate the response to the stimulation. Recently, Frangos et al. [27] have observed in humans that transcutaneous stimulation of the* cymba conchae*, that is, ABVN, produces activation of the classical centers of vagal projections (increased ipsilateral activity in NTS and bilateral activity of spinal nuclei of the trigeminal nerve, the dorsal raphe nucleus, locus coeruleus, contralateral parabrachial area, amygdala, and nucleus accumbens, as well as bilateral activation of the paracentral lobe). Importantly, the electrical stimulation of NTS [40, 41] and some others of these nuclei [4, 42, 43] interferes with epileptogenesis.

According to Vonck et al. [4], Evans et al. [30], and de Lartigue [44], the effects of transcutaneous stimulation of ABVN are due to the stimulation of afferent A and B fibers. Typically, A and B nerve fibers are myelinated and serve mainly mechanical purposes, including touch and sensitivity [45–47]. In our study, a variable percentage of MBP+ nerve fibers were observed in both the* cavum conchae* and MAEC, whose density paralleled that of the nerve profiles. On the other hand, the percentages of myelinated nerve fibers we have found seem to be higher than those reported by Gupta et al. [28] in the ABVN which strongly suggests that nerves innervating the cavum conchae and MAEC other than ABVN also contribute with a variable proportion of A nerve fibers to supply these auricular zones. In addition, since all nerves supplying the auricula and MAEC contain myelinated fibers, all of them may be activated during transcutaneous stimulation, but it remains to be established what quality of sensibility (mechanical, thermal, or chemical) is the most effective in activating these fibers. Studies are in progress in our laboratory to characterize the nerve fibers supplying the cavum conchae and MAEC on the basis of their expression of several ion channels related to different sensory modalities.

## Supplementary Material

Nerve profiles in internal and external segments of MAEC displayed positive immunostaining for NFP (Figs. S1) or S100P (Fig. S3) were observed both subcutaneous and out of the cartilage (Fig. S2). Importantly, most of the nerve profiles identified were placed far off the cutaneous surface isolated from the surrounding tissues even within the cartilage (Fig. S3).
