# Supplementary material for: Innervation of the Human Cavum Conchae and Auditory Canal: Anatomical Basis for Transcutaneous Auricular Nerve Stimulation
Source: Biomed Res Int. 2017 Mar 15;2017:7830919. doi: 10.1155/2017/7830919 (PMC5371220; doi:10.1155/2017/7830919)
Supplement: Supplementary file 1 — Nerve profiles in internal and external segments of MAEC displayed positive immunostaining for NFP (Figs. S1) or S100P (Fig. S3) were observed both subcutaneous and out of the cartilage (Fig. S2). Importantly, most of the nerve profiles identified were placed far off the cutaneous surface isolated from the surrounding tissues even within the cartilage (Fig. S3). [file 7830919.f1.docx]

**Supplementary material**

**Figure S1**.- Serial sections of external segment of MAEC stained with trichromic Masson’s (**a,c**) and immunostained for demonstration of neurofilaments (**b,d**). b is an enlarged detail of square in a. Arrows indicate nerve profiles. Scale bar is identical for a,c and d.

**Figure S2**.- Serial sections of internal segment of MAEC stained with trichromic Masson’s (**a,c**) and immunostained for demonstration of neurofilaments (**b,d**). Arrows indicate nerve profiles. Scale bar is identical for a-d.

**Figure S3**.- Immunohistochemical detection of neurofilaments (a,b) and S100 protein (c) in the external segment of meatus acusticus externus cartilaginous. b is a detail of a (square). Scale bar is identical for b and c.

**
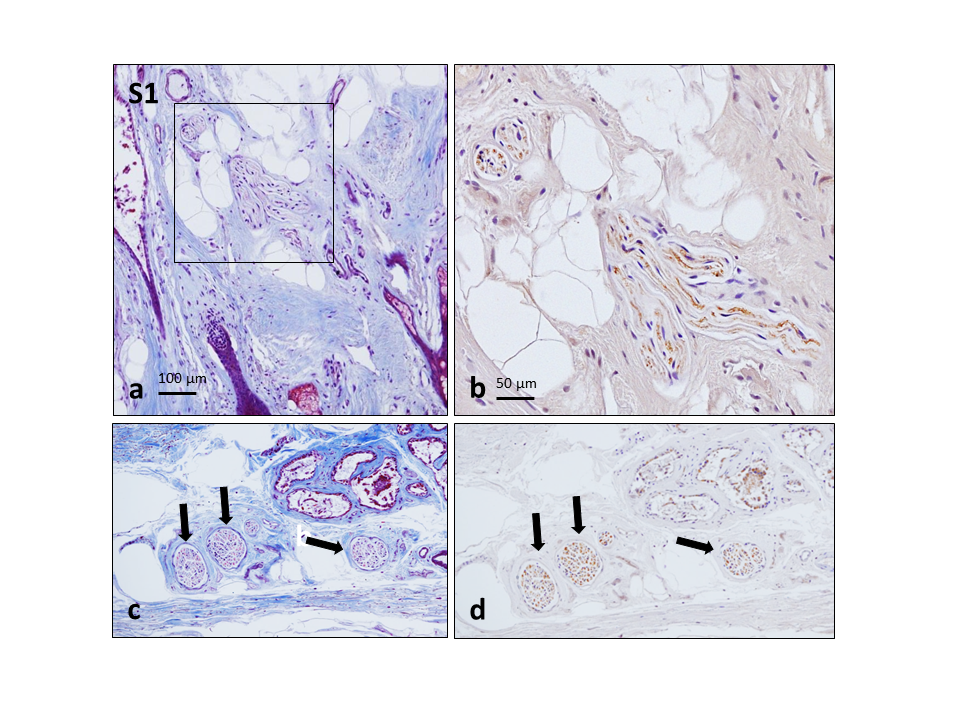
**

**
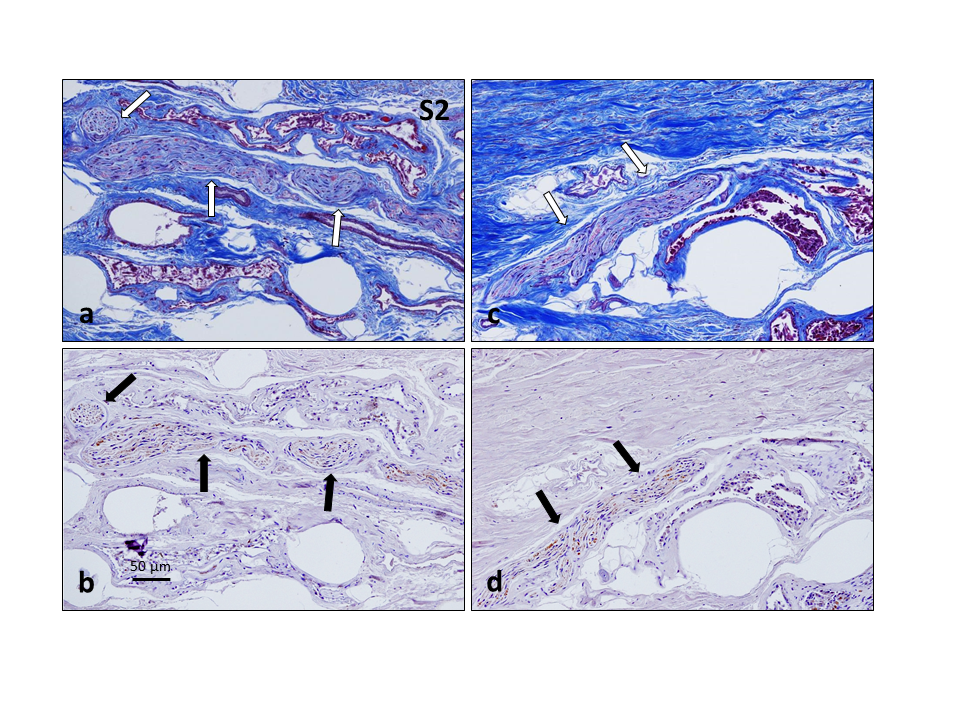
**

**
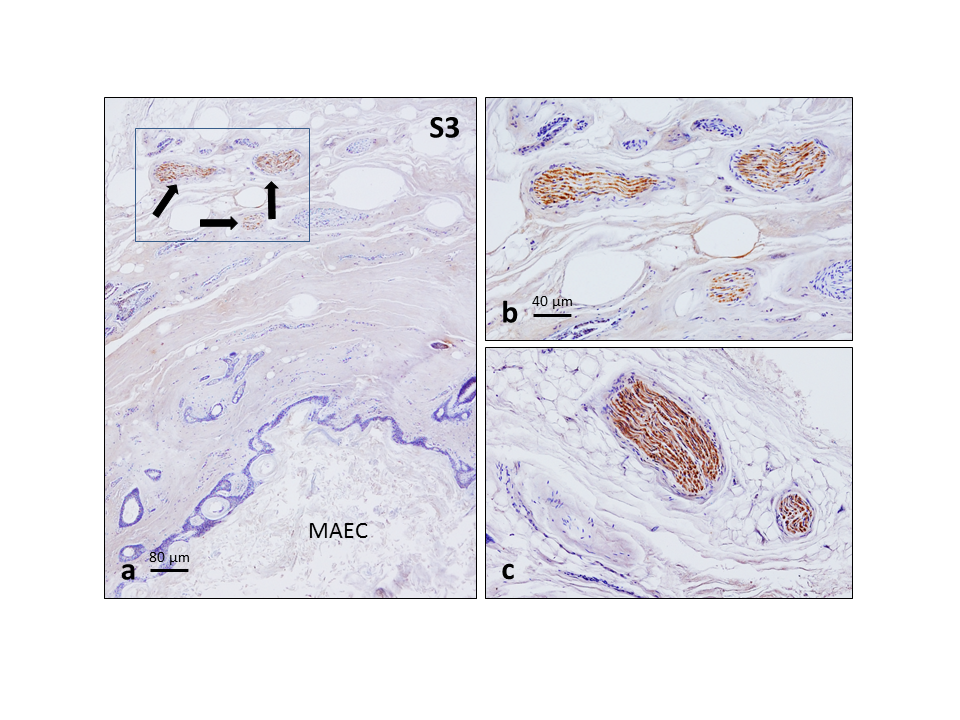
**
